# The near-optimal adjustment of carbon and nitrogen allocations into different organs in early-season rice cultivars with drastically different yield components under nitrogen application

**DOI:** 10.3389/fpls.2025.1537378

**Published:** 2025-02-03

**Authors:** Wen Ning, Lin Su, Dandan Shi, Meina Ji, Xiang Ouyang, Qingfeng Song, Caihong Shao, Xin-Guang Zhu, Shuoqi Chang

**Affiliations:** ^1^ Longping Branch, College of Biology, Hunan University, Changsha, China; ^2^ State Key Laboratory of Hybrid Rice, Hunan Hybrid Rice Research Center, Changsha, China; ^3^ National Key Laboratory of Plant Molecular Genetics, Chinese Academy of Sciences (CAS) Center for Excellence in Molecular Plant Sciences, Shanghai Institute of Plant Physiology and Ecology, Chinese Academy of Sciences, Shanghai, China; ^4^ Institute of Soil Fertilizer and Resources Environment, Jiangxi Academy of Agricultural Sciences, Nanchang, China

**Keywords:** early-season rice, photosynthesis, ^13^C labeling, carbon and nitrogen accumulation, grain yield (GY)

## Abstract

**Introduction:**

Optimized photosynthesis and transport of photosynthate from the upper three leaves in a rice plant is critical for yield formation in rice.

**Methods:**

In this study, we selected two high-yielding early-season rice cultivars, i.e. a large-panicle inbred rice Zhongzao39 (ZZ39) and a plural-panicle hybrid rice Lingliangyou268 (LLY268) with high effective panicle number, to study the translocation of photosynthate from the flag and the basipetal 2^nd^ leaves to the other organs under different nitrogen application scenarios. ^13^CO_2_ labeling was study the proportion of newly assimilated carbon partitioned into different organs.

**Results:**

Results demonstrate that the ratio that ^13^C assimilated in the flag leaves and the basipetal 2^nd^ leaves, and the distribution ratio ^13^C in the organs of ZZ39 and LLY268 cultivars were not affected by nitrogen application. However, at the booting stage, the translocation rate of photosynthate was slower under N150 compared with CK in both flag and the basipetal 2^nd^ leaves labeled with ^13^C. At the grain filling stage, an average of 51% of photosynthetic products labeled with 13C was translocated to the panicle in both cultivars under CK treatment; in contrast, only 43% of leaf photosynthate was translocated to panicles in the N150 treatment. At maturity, the photosynthate labeled with ^13^C distribution ratio in the panicle was greater in the basipetal 2^nd^ leaves than in the flag leaves for ZZ39, whereas the opposite was observed in LLY268. These different photosynthate allocation patterns and their responses to nitrogen application were linked with their corresponding tiller number and number of grains per panicle.

**Discussion:**

This study shows that early-season rice has the ability to flexibly adapt their carbon and nitrogen allocation patterns to gain optimized yield components for higher yield under different nitrogen status. Early season rice can be used as a model system to study the growth strategy selection of plants to changing environment conditions.

## 1 Introduction

Rice is a major staple food for most of Asia countries, which accounts for 60% of the dietary intake in China (Muthayya et al., 2014; Jiang et al., 2019). Double-season rice cropping system is one of the principal rice production models in the East and Southeast Asia. Double-season rice production model includes an early-season rice plantation and a late-season rice plantation; such production model accounts for about 30% of the rice production and 35% of the rice planting area (These data were extracted from the National Bureau of Statistics of China, detail information on the official website: http://www.stats.gov.cn/tjsj/ndsj/). Early-season rice growth period is usually short in South China production areas with the precipitation, light, and other weather conditions mostly unfavorable (Sun et al., 2018), and the pests and diseases being frequent threats (Han et al., 2018). Under such conditions, to be productive, early-season rice is expected to have high flexibility in adjusting their source, sink and flow related activities (Li et al., 2018b; Liang et al., 2023). Comprehensive analysis of the flexibility of early rice source-sink and flow under varying environmental and nitrogen conditions, along with a knowledge of these traits and their adjustments, can provide insights for breeding high-yield, more adaptable rice cultivars.

Nitrogen is a required element for rice growth and development, and is a major determinant of photosynthetic capacity (Ji et al., 2023). Transport of nitrogen compounds can directly affect the allocation of photosynthate (Wang et al., 2022; Liao et al., 2023). Different rice cultivars show different sensitivity to nitrogen fertilizer application (Srikanth et al., 2023). In some rice cultivars, under low nitrogen levels, plants show induced leaf senescence, decreased grain filling period, and enhances the translocation of photosynthates (Li et al., 2018a; Zakari et al., 2020). In some other cultivars, less nitrogen transport from the root, stem and sheath to panicle, and less photosynthates translocation (Dong et al., 2009). These different responses represent different strategies that plants used to adjust their growth and development patterns to optimize their growth fitness. Studying responses of plants under different nitrogen fertilizer conditions therefore represents an effective method to gain insights on ability of plants to acclimate to different environments.

One of the major questions regarding photosynthate translocation is how re-allocation of photosynthate from different leaves to the final yield formation regulated. Generally, the leaves of rice contribute 60% to 80% of photosynthetic products to grain (Zhang et al., 2015; Xu et al., 2020; Wei et al., 2023). Compared to the relatively high volume of research on the impacts of different morphology and physiological characteristics of leaves on rice yields (Wang et al., 2016; Gao et al., 2021; Ding et al., 2022), however, there are very few studies on the dynamics of translocation and distribution of photosynthetic products from the upper trifoliate leaves of the canopy to various organs (Ling et al., 1982). The studies on allocation of photosynthates from leaves, especially the flag leaves and basipetal 2^nd^ leaves, are relatively scarce. Systematic studies are required to examine the process of photosynthate synthesis, transport and allocation between different organs, especially from leaves to other organs, in particular, on how re-allocation respond to different environments and their potential impacts on productivity.

This study utilizes two cultivars: ZZ39 (inbred rice) and LLY268 (hybrid rice) to study responses of source, sink and flow related activities to different nitrogen application levels. We chose these two cultivars since they are representative early season rice cultivars, extensively cultivated in Hunan Province China, and show high photosynthetic rates and greater yields (Li et al., 2023). The aim of this study was to studying their strategies to adjust their growth and developmental patterns under different conditions, through examining their changes in the allocation of photosynthate from the flag leaves and basipetal 2^nd^ leaves to other organs under different nitrogen application levels.

## 2 Materials and methods

### 2.1 Test site and test cultivars

The experiment was conducted in the Experimental Base of the State Key Laboratory of Hybrid Rice in Changsha, Hunan Province, China, located at coordinates 28°11′46.97″N 113°05′4.54″E. The soil has a pH of 6.02, a concentration of fast-acting phosphorus of 23.8 mg·kg^-1^, a concentration of fast-acting potassium of 90.0 mg·kg^-1^, a concentration of alkaline nitrogen of 197.5 mg·kg^-1^, and an organic matter content of 21.3 g·kg^-1^. We used Zhongzao39 (ZZ39), an *indica* inbred rice cultivar, and Lingliangyou268 (LLY268), an *indica* hybrid rice. ZZ39, passed China’s validation in 2012, with an average of 112.2 days of full-life span, the production experiment in 2011 showed a yield of 7855 kg·hm^-2^ (https://www.ricedata.cn/variety/varis/605891.htm). LLY268, the maternal cultivar is Xiangling628, the paternal cultivar is Hua268, passed China’s validation in 2008, with an average of 112.2 days of full-life span, the production experiment in 2007 showed a yield of 7720 kg·hm^-2^ (https://www.ricedata.cn/variety/varis/605259.htm).

### 2.2 Experimental design

The experiment included two cultivars of ZZ39 and LLY268, together with two different nitrogen application procedures. The experimental treatments included the application of 150 kg·hm^-2^ of nitrogen (N150), a control group with no nitrogen fertilizer (CK). Nitrogen was applied in three stages: basal fertilizer (two days before transplantation), tillering fertilizer (7 days after transplanting), and panicle fertilizer (during the fourth stage of panicle initiation), the nitrogen application rate followed a ratio of 6:3:1 for basal fertilizer, tillering fertilizer and panicle fertilizer. Nitrogen-based fertilizer, specifically urea, was used. All treatments were treated with 90 kg·hm^-2^ of superphosphate (containing P_2_O_5_ 13.5%), which was applied as basal fertilizer at one time (two days before transplantation). A total of 180 kg·hm^-2^ of potassium chloride, with a K_2_O content of 52%, was applied, the ratio of basal fertilizer to panicle fertilizer was 1:1.

The plant was cultivated in plastic pots of 34 cm in diameter and 30 cm in height. Each pot contained 14.0 kg of paddy soil and was fertilized according to the recommended dosage for the potting region. Conversion according to Lu (Lu et al., 2016), nitrogen (N) was applied at 1.36 g/pot, superphosphate (P_2_O_5_) was applied at 0.83 g/pot, potassium chloride (K_2_O) was applied at 1.63 g/pot. A total of 48 pots were set up for the entire experiment with 12 pots per treatment, i.e. 48 replications per treatment. The seeds were planted on March the 30^th^, 2023, and moved to the pots on April the 29^th^. Each pot contains 4 holes, with 3 seedlings placed in each hole. The rice plants were well-irrigated, weed controlled, pest controlled. These plants were used for the later experiments. These rice lines were headed on June the 19^th^, and finally harvested on July the 17^th^. The measurements were conducted on completely expanded mature leaves.

### 2.3 ^13^CO_2_ labeling experiments

**Figure 1:**
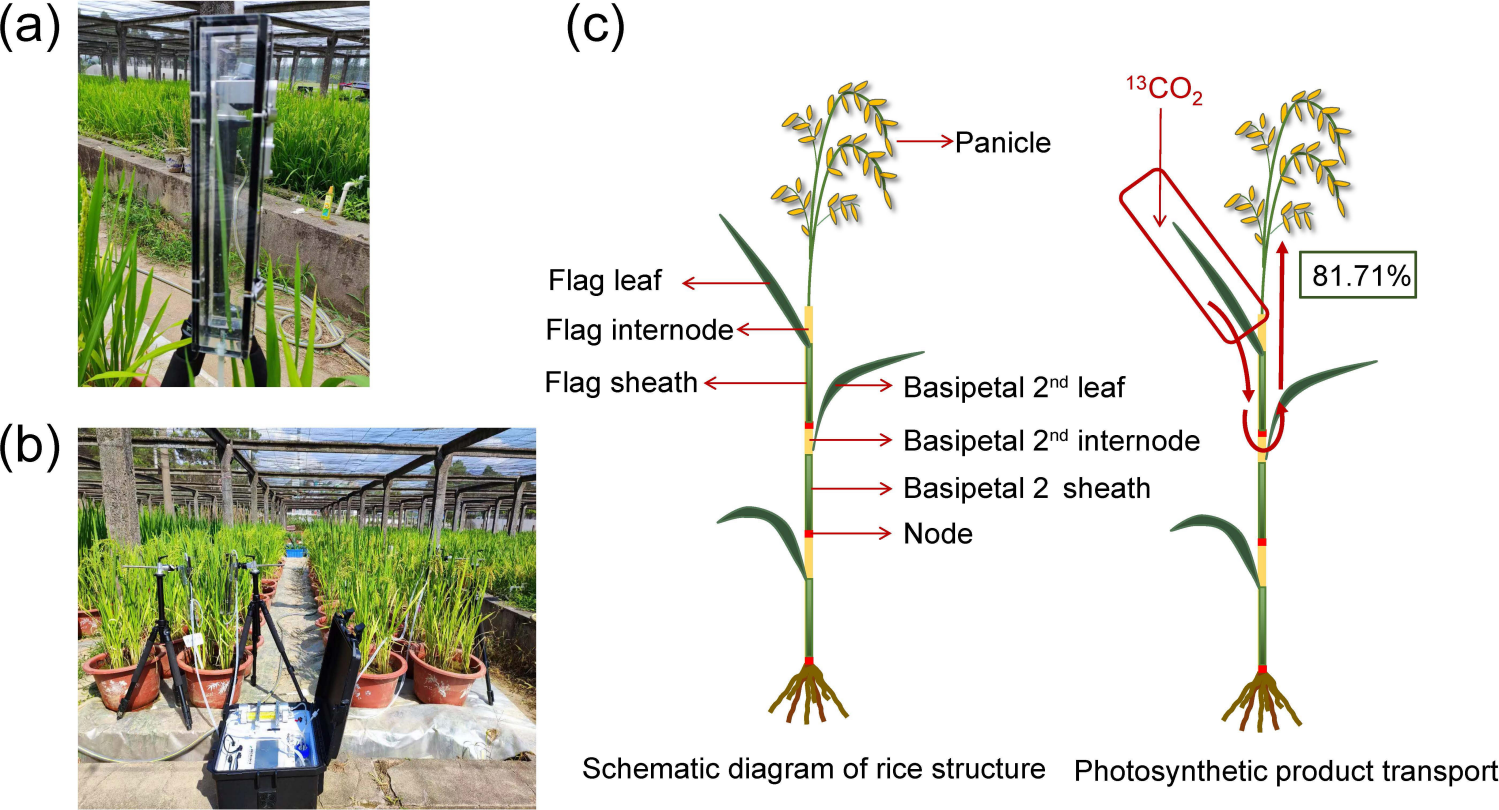
Photos of the custom-built ^13^CO_2_ labeling system. **(A)** transparent leaf chamber, **(B)** ^13^CO_2_ continuous labeling equipment. **(C)** schematic diagram of rice structure and photosynthetic product translocation.

The labeling procedure follows Xiao (Xiao et al., 2019). Leaves were placed in a transparent leaf chamber measuring 50 cm×5 cm×5 cm (**Figure 1A**). The chamber was continuously supplied with ^13^CO_2_ using a device specifically designed for continuous labeling (**Figures 1B, C**). The ^13^CO_2_ was produced by mixing a solution of 0.2 mol·L^-1 13^C-Na_2_CO_3_ (with a 99% abundance of ^13^C-Na_2_CO_3_) with 1 mol·L^-1^ hydrochloric acid (HCl). The labeling process lasted for 3 hours, from 9:00 to 12:00 AM on a sunny day. Each treatment was labeled three times to ensure accuracy of the results. The labeling was conducted at the rice booting stage (May the12^th^ to May the 18^th^, 2023), with each treatment being labeled in three separate repetitions. Three unlabeled rice pots were simultaneously used to evaluate the natural abundance of ^13^C in plants. The detailed information on the position of ^13^C labeled leaves and the time at which they were sampled for each treatment are shown in **Table 1**.

**Table 1:** ^13^C labeling leaf position and sampling stage.

Cultivar		N Treatment	Labeled Leaf	Labeling and sampling stage			
				Labeling stage	First sampling	Second sampling	Third sampling
ZZ39	CK		Flag leaf	Booting	Booting	Grain filling	Maturity
			Basipetal 2^nd^ leaf				
	N150		Flag leaf				
			Basipetal 2^nd^ leaf				
LLY268	CK		Flag leaf				
			Basipetal 2^nd^ leaf				
	N150		Flag leaf				
			Basipetal 2^nd^ leaf				

### 2.4 Measurement and methods

#### 2.4.1 Measurement of carbon and nitrogen concentrations

Six individual stems (tillers) showing consistent growth in each treatment were chosen. The sheaths, leaves, and internodes of these stems were separated into different categories (flag leaf, basipetal 2^nd^ leaf, basipetal 3^rd^ leaf, flag sheath, basipetal 2^nd^ sheath, basipetal 3^rd^ sheath, flag internode, basipetal 2^nd^ internode, basipetal 3^rd^ internode, and panicle). The samples were then subjected to a 30-minute treatment at 105°C to kill any living organisms, followed by drying at 80°C until a constant weight was achieved. The biomass was measured by weighing the samples after they were crushed and mixed. The resulting mixture was then passed through an 80-mesh sieve. The carbon and nitrogen concentrations of each organ were measured using an elemental analyzer (Vario Marco cube, Elementar, Germany). The carbon and nitrogen contents of a single organ were then computed by multiplying the respective concentrations with the dry matter weight of the samples. Here is the formula:


(1) $$\begin{array}{c} C_{\text{s}}=W_{\text{s}}\times C_{\text{c}} \end{array}$$

(2) $$\begin{array}{c} N_{\text{s}}=W_{\text{s}}\times N_{\text{c}} \end{array}$$

where C_s_ and N_s_ refer to total C content and N content of the samples; W_s_ refers to the dry matter weight of the samples, C_c_ and N_c_ refer to the C concentration and N concentration of the samples (mg/g).

#### 2.4.2 ^13^C concentration and dispersion

The ^13^C abundance of the samples was determined using a stable isotope mass spectrometer (DELTA V Advantage, Thermo, USA). The ^13^C abundance is defined as the ratio of the number of moles of ^13^C in the sample to the number of moles of all carbon isotopes. The samples’ fixed ^13^C content and the distribution ratio of ^13^C in the organs were estimated according to the method of Zhu (Zhu et al., 2017). Here is the formula:


(3) C 13s=[AL−AUL]×Cs

The variable ^13^C_s_ represents the total ^13^C content of the samples, whereas A_L_ and A_UL_ represent the ^13^C atoms% in the labeled and unlabeled samples, respectively.

The ^13^C content of the single stem was obtained by summing the ^13^C content of each organ. Here is the formula:


(4) C 13stem=∑k=0nC 13s

where ^13^C_stem_ is the total ^13^C content of single stem.

The distribution ratio of ^13^C in the organ is determined using the following calculation.


(5) C 13r=13Cs/13Cstem×100%

where ^13^C_r_ is the distribution ratio of samples, ^13^C_s_ is the total ^13^C content of samples, respectively.

#### 2.4.3 Leaf morphology and photosynthetic rate

Leaf area was quantified by measuring the length and width of three leaves in the canopy with a portable leaf area meter (Ci-203, CID bio-science inc, USA). Measurements were conducted at the tillering stage (May the 22^nd^) and panicle initiation stage (June the 4^th^) with six replications.

Leaf thickness was measured with a hand-held digital leaf thickness tester (YHT100195) with a precision of 0.001 mm/0.00005. Ten points were taken evenly from the middle of the leaf to the tip of the leaf (5cm), excluding the main vein. The average value of these measurements was considered as the leaf thickness of the leaf during the tillering stage (May the 22^nd^) and panicle initiation stage (June 4^th^) with six replications for each treatment.

Chlorophyll content was assessed during the tillering stage (May the 22^nd^) and panicle initiation stage (June the 4^th^) by measuring SPAD values using a portable chlorophyll meter (SPAD 502, Konica Minolta Optics, Japan). Measurements were taken at five equidistant points along the leaf, starting from the middle and ending 5 cm from the leaf tip. The main veins were avoided, and the average SPAD value was used to represent the chlorophyll content of the leaf with six replications.

The saturated net photosynthetic rate was quantified in the upper and middle regions of the leaves using a Li-6800 portable photosynthesis analyzer (Li-Cor Inc., Lincoln, NE, USA) on a sunny day between 9:00 and 11:00. This measurement was conducted during the panicle initiation stage (June the 4^th^) and the grain filling stage (June 27^th^). The temperature in the leaf chamber (6 cm^2^) was set to 28°C, the relative air humidity was set to 60%, the light intensity was set to 1600 μmol·m^-2^·s^-1^, and the CO_2_ concentration was set to 400 μmol·m^-2^·s^-1^. We used six replications for these measurements.

#### 2.4.4 Agronomic traits and yield components

The tiller number was measured during the panicle initiation stage (June the 4^th^), while the count of effective panicles was conducted during the maturity stage. A total of 15 replications were recorded for each treatment.

Plant height: At maturity stage (July the 17^th^), the height of the tallest tiller plant in each hole was measured from the soil surface to the highest point of the tiller. This measurement was considered as the height of the rice plant in that hole, with six replications.

Dry matter weight and yield components were assessed by selecting six representative, pest and disease-free rice plants from each treatment after maturity (July the 17^th^). The living tissues of plants were killed at 105°C and then dried at 80°C until a constant weight was achieved. The dry matter weight was measured, and the number of grains per panicle, seed-setting rate, thousand-grain weight, and yield were determined through seed testing.

The weight of individual panicle was measured by sampling single stem at the stages of booting (June the15^th^), grain filling (June the 27^th^) and maturity (July the17^th^). The stems were then dried at a temperature of 80°C until a constant weight was achieved, after which they were weighed following the separation of the individual stem organs.

The panicle growth rate is calculated as the difference in panicle weights between two reproductive periods, divided by the time difference. For each treatment, seven replications were chosen.

### 2.5 Statistical analysis

The data were analyzed and assessed for statistical significance using Microsoft Excel 2021 and SPSS 26.0. GraphPad Prism 9 and Origin 2022 were utilized for creating plots.

## 3 Results and analyses

### 3.1 The yield and yield components of two super early-season rice cultivars under nitrogen application

At maturity stage, following the N150 treatment, the grain yields of ZZ39 and LLY268 increased by 90.42% and 72.61% respectively compared to CK. Additionally, the N150 treatment resulted in a considerable increase in the number of panicles for both ZZ39 and LLY268 cultivars. More precisely, the number of panicles for ZZ39 showed a significant increase of 58.80%, while for LLY268, the increase was 27.78%. In addition, the N150 treatment resulted in a 48.49% increase in the number of grains per panicle for LLY268, and a 16.4% increase for ZZ39. Under N150 treatment, the seed-setting rate of ZZ39 increased significantly compared to CK by 10.0%, but there was no significant difference for LLY268. Nevertheless, there was no substantial difference between the two treatments in the thousand-grain weight of the two cultivars (**Table 2**).

**Table 2:** The yield and yield components of super early-season rice.

Cultivar	Treatment	Effective panicles per plant	Grain number per panicle	Seed-setting rate (%)	1000-grain weight (g)	Grain yield (g plant^−1^)
ZZ39	CK	4.83 ± 0.41 b	130.53 ± 12.45 b	81.95 ± 3.96 b	23.71 ± 0.74 a	12.95 ± 1.97 b
	N150	7.67 ± 1.38 a	151.94 ± 5.71 a	87.55 ± 2.18 a	23.79 ± 0.68 a	24.66 ± 2.95 a
LLY268	CK	9.00 ± 0.89 b	79.97 ± 9.10 b	83.03 ± 5.05 a	21.05 ± 1.17 a	14.75 ± 2.65 b
	N150	11.50 ± 1.52 a	118.75 ± 14.50 a	85.32 ± 4.45 a	22.16 ± 1.26 a	25.46 ± 3.75 a

Different letters show significant differences between nitrogen applications at 5% probability level.

### 3.2 The agronomic characteristics of two super early-season rice cultivars under nitrogen application

**Figure 2:**
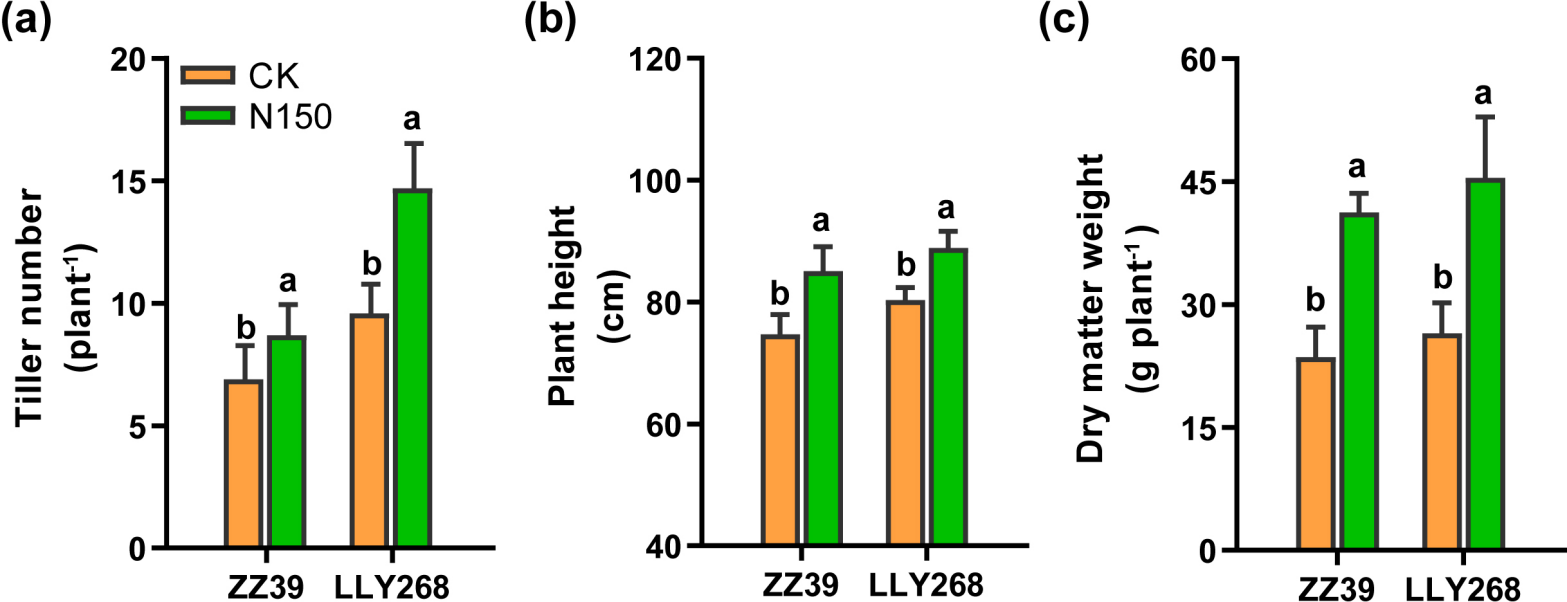
Agronomic traits of early-season rice under different nitrogen fertilizer applications. **(A)** tiller number at the stage of panicle initiation (June 6^th^), **(B)** plant height at maturity (July 17^th^), **(C)** dry matter weight at maturity (July 17^th^). Different letters show significant differences between nitrogen applications at 5% probability level. 15 replications of tiller numbers were measured for each treatment. 6 replicates of plant height and dry matter weight were measured.

At the stage of panicle initiation, we studied the number of tillers in ZZ39 and LLY268, and measured the plant height and dry mass of both cultivars at the maturity stage. The results indicate a significant increase in the number of tillers, plant height, and dry matter weight of rice in the N150 treatment (**Figure 2**). Specifically, the number of tillers for ZZ39 and LLY268 increased by 26.09% and 53.13% (**Figure 2A**), while plant height increased by 13.89% and 10.67% (**Figure 2B**). Additionally, the dry matter weight increased by 74.71% and 71.62% in the N150 treatment respectively, compared to CK (**Figure 2C**).

### 3.3 The leaf morphological traits in two super early-season rice cultivars

**Figure 3:**
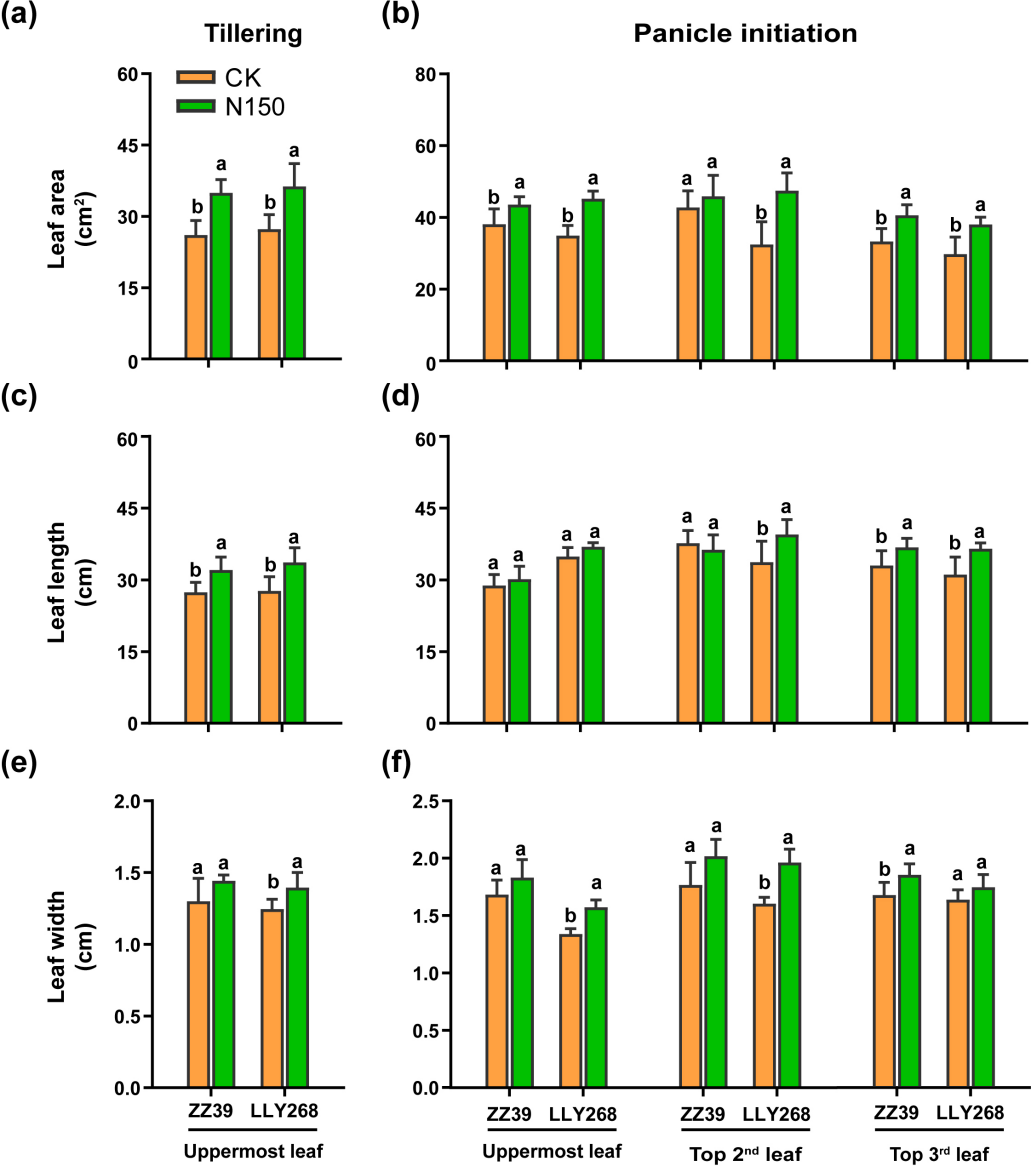
Leaf morphology of canopy upper three leaves of early-season rice under different nitrogen applications. **(A, B)** leaf area, **(C, D)** leaf length, **(E, F)** leaf width. Different letters show significant differences between nitrogen applications at 5% probability level. Tillering stage data was measured on May 22^nd^, panicle initiation stage date was measured on June 4^th^. 6 replications were measured for each treatment.

During the tillering and panicle initiation stages, we measured leaf length, width, area (**Figure 3**), and thickness (**Table 3**). Both cultivars showed increased leaf length and leaf widths when exposed to the N150 treatment, as compared to CK. At the tillering stage, the leaf areas of the uppermost leaves for ZZ39 and LLY268 increased by 33.93% and 33.13% respectively, compared to CK. In addition, the total leaf areas of the top three leaves of ZZ39 and LLY268 increased by 13.97% and 34.53% respectively at the panicle initiation stage, compared to CK, under N150 treatment. Nevertheless, there was no notable difference in the thickness of the leaves between the two cultivars when subjected to N150 treatment, as compared to CK, during the tillering and panicle initiation stages (**Table 3**).

**Table 3:** Effect of nitrogen application on leaf thickness of super early-season rice (mm).

Cultivar	N Treatment	Tillering	Panicle initiation		
		Uppermost leaf	Upmost leaf	Top 2^nd^ leaf	Top 3^rd^ leaf
ZZ39	CK	0.165 ± 0.018 a	0.193 ± 0.008 a	0.176 ± 0.022 a	0.178 ± 0.013 a
	N150	0.163 ± 0.012 a	0.191 ± 0.022 a	0.198 ± 0.017 a	0.171 ± 0.008 a
LLY268	CK	0.136 ± 0.005 a	0.181 ± 0.013 a	0.162 ± 0.016 a	0.153 ± 0.022 a
	N150	0.143 ± 0.007 a	0.193 ± 0.010 a	0.173 ± 0.036 a	0.175 ± 0.006 a

Different letters show significant differences between nitrogen applications at 5% probability level. Tillering stage data was measured on May 22, panicle initiation stage date was measured on June 4. 6 replications were measured for each treatment.

**Figure 4:**
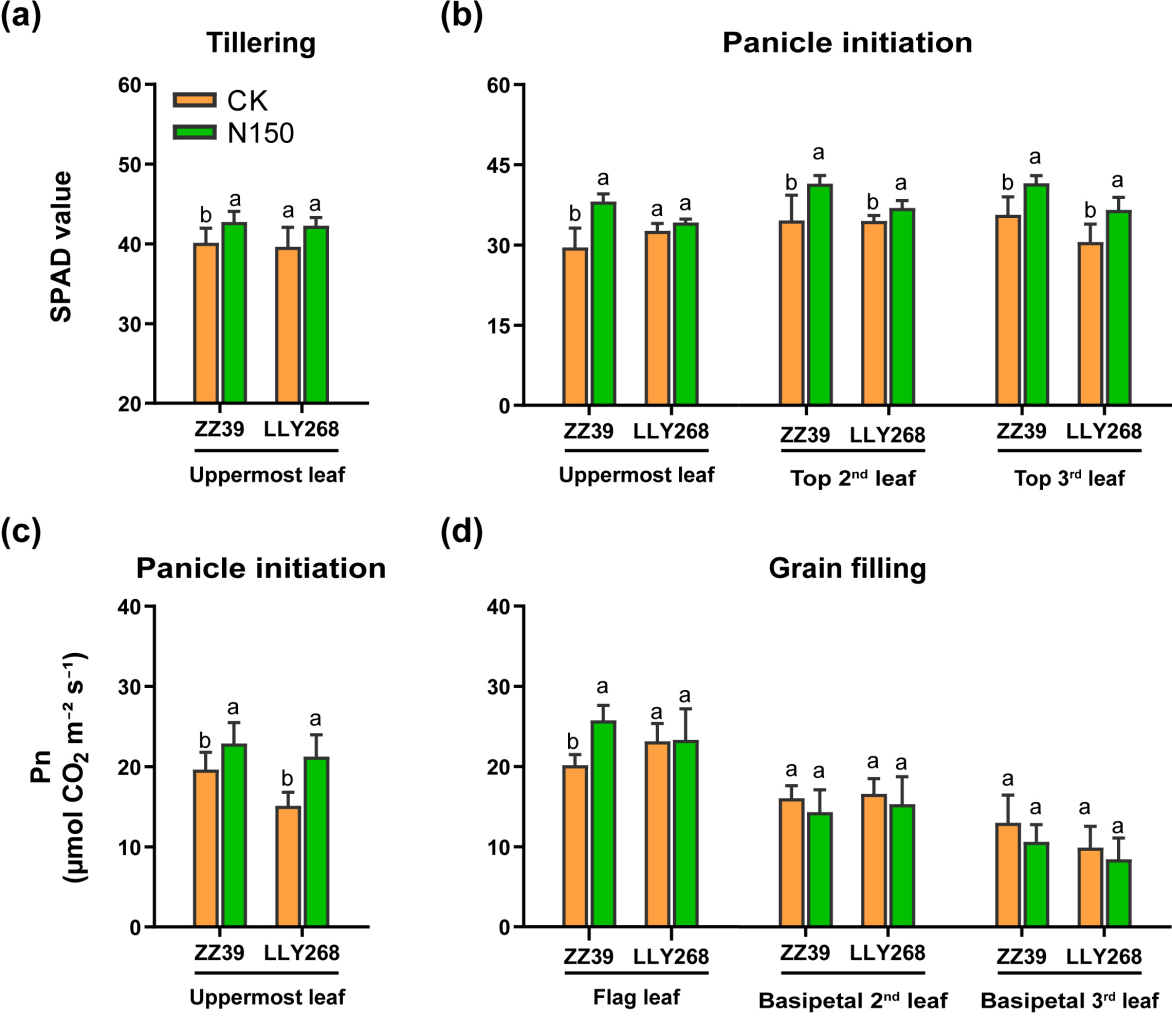
Photosynthetic physiology of canopy basipetal three leaves of super early-season rice leaves under different nitrogen applications. **(A, B)** SPAD value; **(C, D)** net photosynthetic rate (Pn). Different letters show significant differences between nitrogen applications at 5% probability level. Tillering stage data was measured on May 22^nd^, panicle initiation stage date was measured on June 4^th^, grain filling stage date was measured on June 27^th^. 6 replications were measured for each treatment.

The leaf SPAD values were measured during the tillering and panicle initiation stages (**Figures 4A, B**), whilst the net saturated photosynthetic rate was measured during the panicle initiation and grain filling stage (**Figures 4C, D**). Both cultivars showed an increased SPAD values when subjected to the N150 treatment, as compared to the CK. At the tillering stage, the SPAD value of the upmost leaf of ZZ39 and LLY268 increased by 6.53% and 6.69% under the N150 treatment, respectively, compared to CK (**Figure 4A**). In comparison to the CK (**Figure 4B**), the total SPAD values of the upper three leaves in the canopy of ZZ39 and LLY268 under N150 treatment increased by 21.43% and 10.15% respectively at panicle initiation stage. Moreover, the topmost leaf of ZZ39 showed an 16.78% increase in its saturation net photosynthetic rate, while LLY268 showed a 40.73% increase, under the N150 treatment during the panicle initiation stage. Furthermore, during the grain filling stage, only the highest leaf of ZZ39 exhibited a noteworthy increase of 27.78% in its saturated net photosynthetic rate following the N150 treatment, as compared to CK (**Figure 4D**).

### 3.4 The nitrogen concentration and nitrogen content in various organs of a single tiller of the two super early-season rice cultivars under nitrogen application

**Figure 5:**
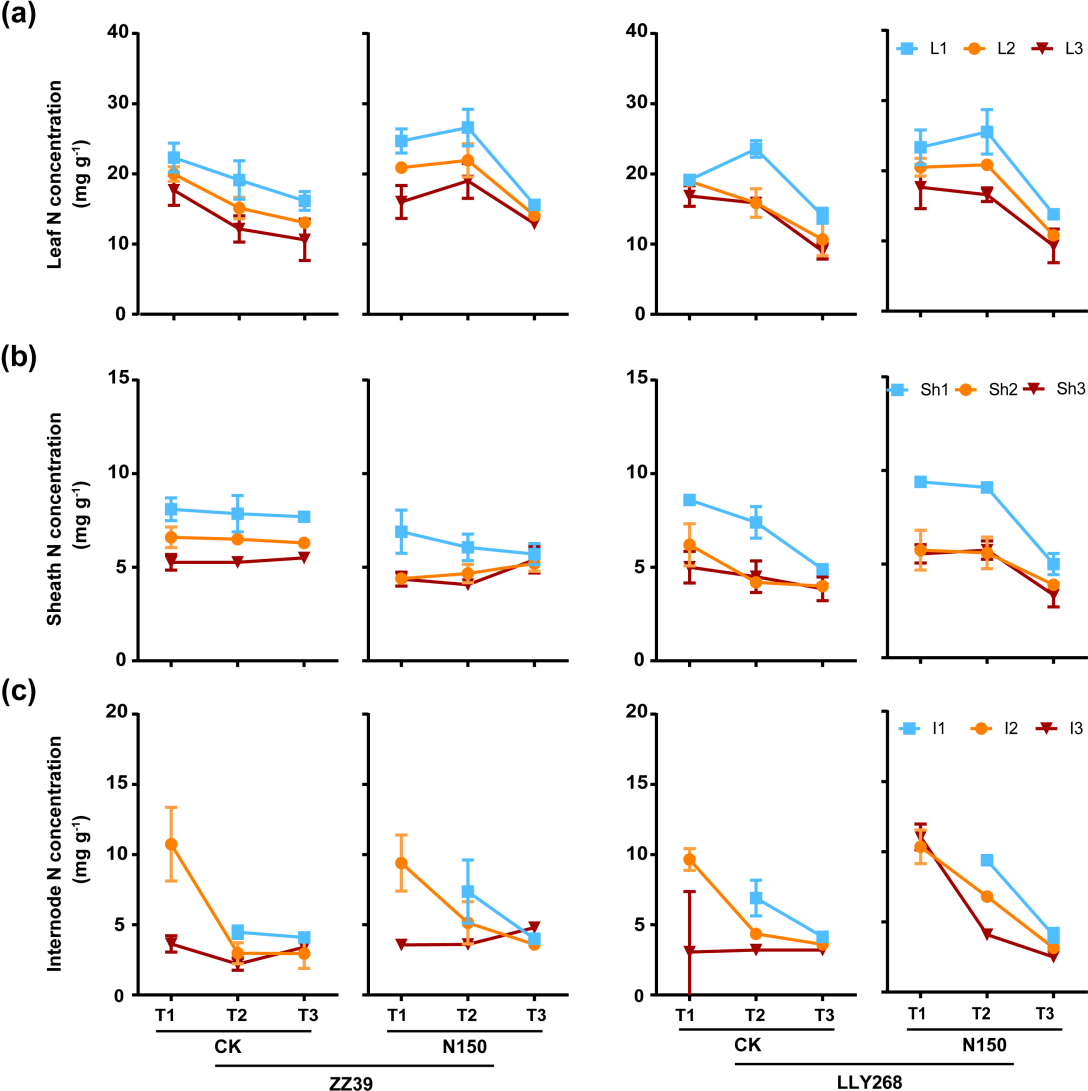
Nitrogen concentration of rice single stem nutrient organs at various stages. **(A)** leaf, **(B)** sheath, **(C)** internode. Note: T1: booting stage, T2: grain filling stage, T3: maturity; L1: flag leaf, L2: basipetal 2^nd^ leaf, L3: basipetal 3^rd^ leaf; Sh1: flag sheath, Sh2: basipetal 2^nd^ sheath, Sh3: basipetal 3^rd^ sheath; I1: flag internode, I2: basipetal 2^nd^ internode, I3: basipetal 3^rd^ internode. The flag internode was not elongated at T1, so no data were available, and the uppermost internode of this stage is I2. Booting stage samples were obtained on June 12^th^ to June 18^th^, the day of the ^13^CO_2_ labeling, grain filling stage samples were obtained on June 27^th^, maturity stage samples were obtained on July 17^th^. 6 replications were measured for each treatment.

An overall pattern of the increase and then decrease of nitrogen (N) concentration in the uppermost leaves(flag leaves)was observed in each treatment of LLY268 and the N150 treatment of ZZ39 from the booting stage to the grain filling stage and to the maturity stage (**Figure 5A**). In contrast, the basipetal 2^nd^ leaves and basipetal 3^rd^ leaves exhibited a consistent trend of decrease or remain constant and then decrease except the N150 treatment of ZZ39, as did the N concentration in the sheaths and internodes (**Figures 5B, C**). The nitrogen concentrations in the leaves, sheaths, and internodes of N150 treatment in the two cultivars did not show a significant difference compared to that of the CK after nitrogen application. There was no noticeable disparity in the concentration of nitrogen (N) between ZZ39 and LLY268 when subjected to the same treatment. Organs located in the top layers of the canopy showed increased nitrogen (N) concentration compared to organs in the lower layers of the canopy.

**Figure 6:**
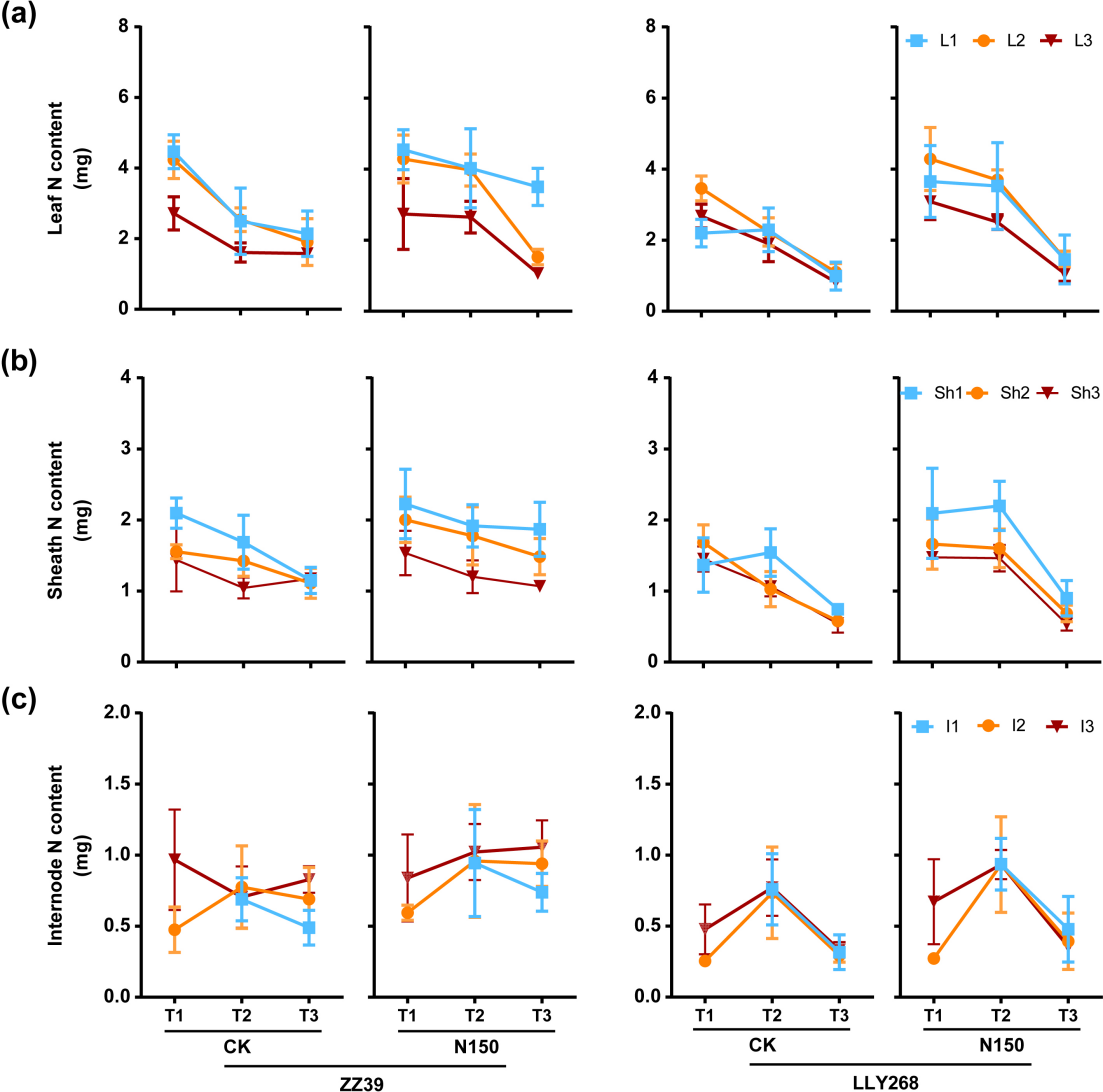
Nitrogen content of rice single stem nutrient organs at various stages. **(A)** leaf, **(B)** sheath, **(C)** internode. Note: T1: booting stage, T2: grain filling stage, T3: maturity; L1: flag leaf, L2: basipetal 2^nd^ leaf, L3: basipetal 3^rd^ leaf; Sh1: flag sheath, Sh2: basipetal 2^nd^ sheath, Sh3: basipetal 3^rd^ sheath; I1: flag internode, I2: basipetal 2^nd^ internode, I3: basipetal 3^rd^ internode. The flag internode was not elongated at T1, so no data were available, and the uppermost internode of this stage is I2. Booting stage samples were obtained on June 12^th^ to June 18^th^, the day of the ^13^CO_2_ labeling, grain filling stage samples were obtained on June 27^th^, maturity stage samples were obtained on July 17^th^. 6 replications were measured for each treatment.

From the booting stage to grain filling stage, the nitrogen content in the upper three leaves of canopy in CK decreased faster than that of N150 treatment in both cultivars, while the N content in the leaves of N150 treatment drop faster than CK from grain filling stage to maturity stage in two cultivars (**Figure 6A**). The N content in upper three sheaths of CK in ZZ39 dropped faster that of N150 treatment in ZZ39 from booting stage to the grain filling stage and maturity stage. The N content of flag sheaths had similar decreasing trend for CK and N150 treatment in LLY268 (**Figure 6B**), and the upper three internode have higher N content in grain filling stage (**Figure 6C**).

### 3.5 The carbon concentration and carbon content in various organs of two super early-season rice cultivars under nitrogen application

**Figure 7:**
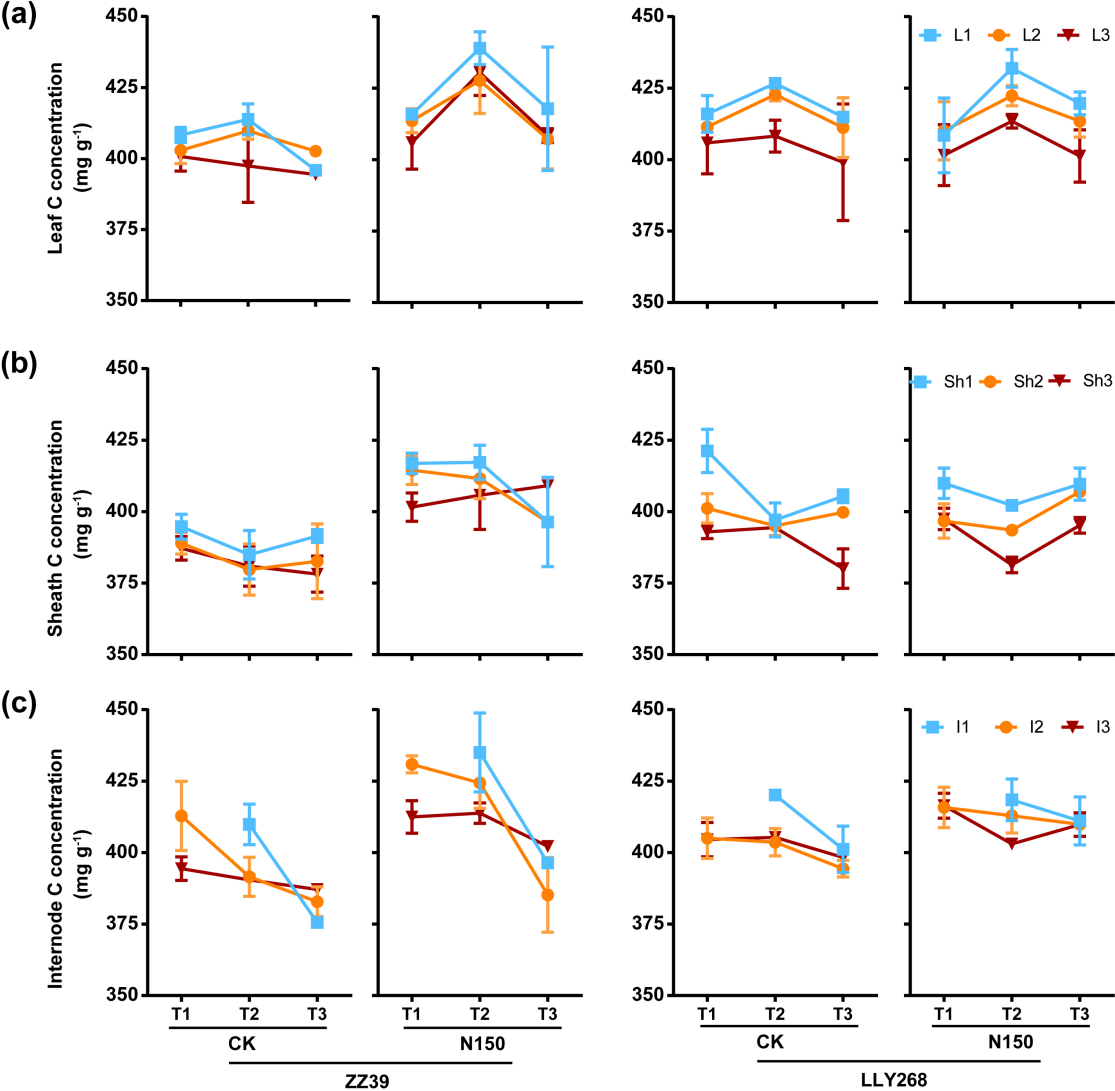
Carbon concentration of rice single stem nutrient organs at various stages. **(A)** leaf, **(B)** sheath, **(C)** internode. Note: T1: booting stage, T2: grain filling stage, T3: maturity; L1: flag leaf, L2: basipetal 2^nd^ leaf, L3: basipetal 3^rd^ leaf; Sh1: flag sheath, Sh2: basipetal 2^nd^ l sheath, Sh3: basipetal 3^rd^ sheath; I1: basipetal 1^st^ internode, I2: basipetal 2^nd^ internode, I3: basipetal 3^rd^ internode. The flag internode was not elongated at T1, so no data were available, and the uppermost internode of this stage is I2. Booting stage samples were obtained on June 12^th^ to June 18^th^, the day of the ^13^CO_2_ labeling, grain filling stage samples were obtained on June 27^th^, maturity stage samples were obtained on July 17^th^. 6 replications were measured for each treatment.

In both treatments of two cultivars, we observed a consistent trend of the carbon concentration changes in leaves, i.e. an initially increase followed by a gradual decrease from the booting to the grain filling and maturity stages (**Figure 7A**). In contrast, the carbon concentration in sheaths exhibited little changes during growth (**Figure 7B**). Following the application of nitrogen, the levels of carbon in the leaves, sheaths, and internodes of the N150 treatment in ZZ39 were increased compared to CK (**Figures 7A–C**). The concentration of carbon (C) in the sheaths and internodes were marginally elevated in LLY268 compared to ZZ39(**Figure 7A, C**), nevertheless, there was no notable difference in carbon (C) concentration between the two cultivars under N150 treatment. The carbon concentration in different tissues in the upper layers of a canopy continuously exceeded that in tissues at the lower layers of a canopy, respectively of their growth locations.

**Figure 8:**
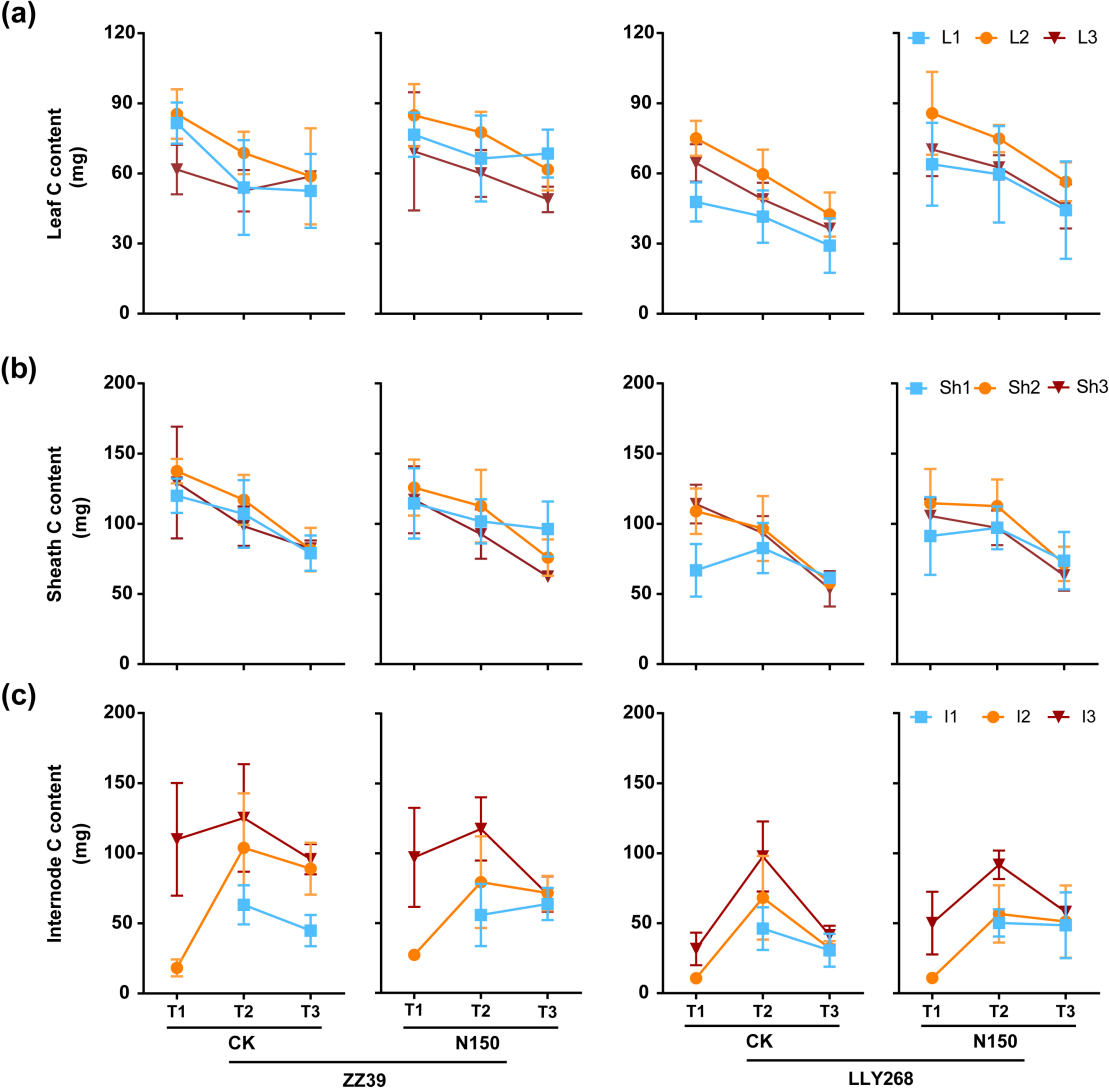
Carbon content of rice single stem nutrient organs at various stages. **(A)** leaf, **(B)** sheath, **(C)** internode. Note: T1: booting stage, T2: grain filling stage, T3: maturity; L1: flag leaf, L2: basipetal 2^nd^ leaf, L3: basipetal 3^rd^ leaf; Sh1: flag sheath, Sh2: basipetal 2^nd^ sheath, Sh3: basipetal 3^rd^ sheath; I1: flag internode, I2: basipetal 2^nd^ internode, I3: basipetal 3^rd^ internode. The flag internode was not elongated at T1, so no data were available, and the uppermost internode of this stage is I2. Booting stage samples were obtained on June 12^th^ to June 18^th^, the day of the ^13^CO_2_ labeling, grain filling stage samples were obtained on June 27^th^, maturity stage samples were obtained on July 17^th^. 6 replications were measured for each treatment.

The carbon (C) content of the leaf and sheaths of both cultivars in each treatment consistently decreased from the booting stage, except to the flag leaves and the basipetal 2^nd^ leaves from the grain filling stage to maturity stage (**Figure 8A, B**). Nevertheless, the carbon content of the internodes had an initial increase from booting stage to grain filling stage, and followed by a subsequent decrease from grain filling stage to maturity stage (**Figure 8C**). As for the carbon content under two treatments, the carbon content in the leaves of LLY268 increased by 17.32% compared to CK at the booting stage. The carbon concentration in sheaths and internodes showed little changes between different treatments at the booting, grain filing and maturity stages. Under the CK treatment, the total carbon content in leaves and sheaths of ZZ39 and LLY268 were reduced by 19.98% and 12.93% respectively. By comparison, the N150 treatment resulted in a decrease of just 12.89% for ZZ39 and 5.96% for LLY268 (**Figure 8A, B**). As for the two rice cultivars, the total carbon contents of leaves, sheaths, and internodes in ZZ39 were higher than that of LLY268 in both experimental circumstances at the booting stage, showing an average increase of 34.35%.

### 3.6 The translocation and partitioning of photosynthetic products from the leaves in several organs of the two super early-season rice cultivars

**Figure 9:**
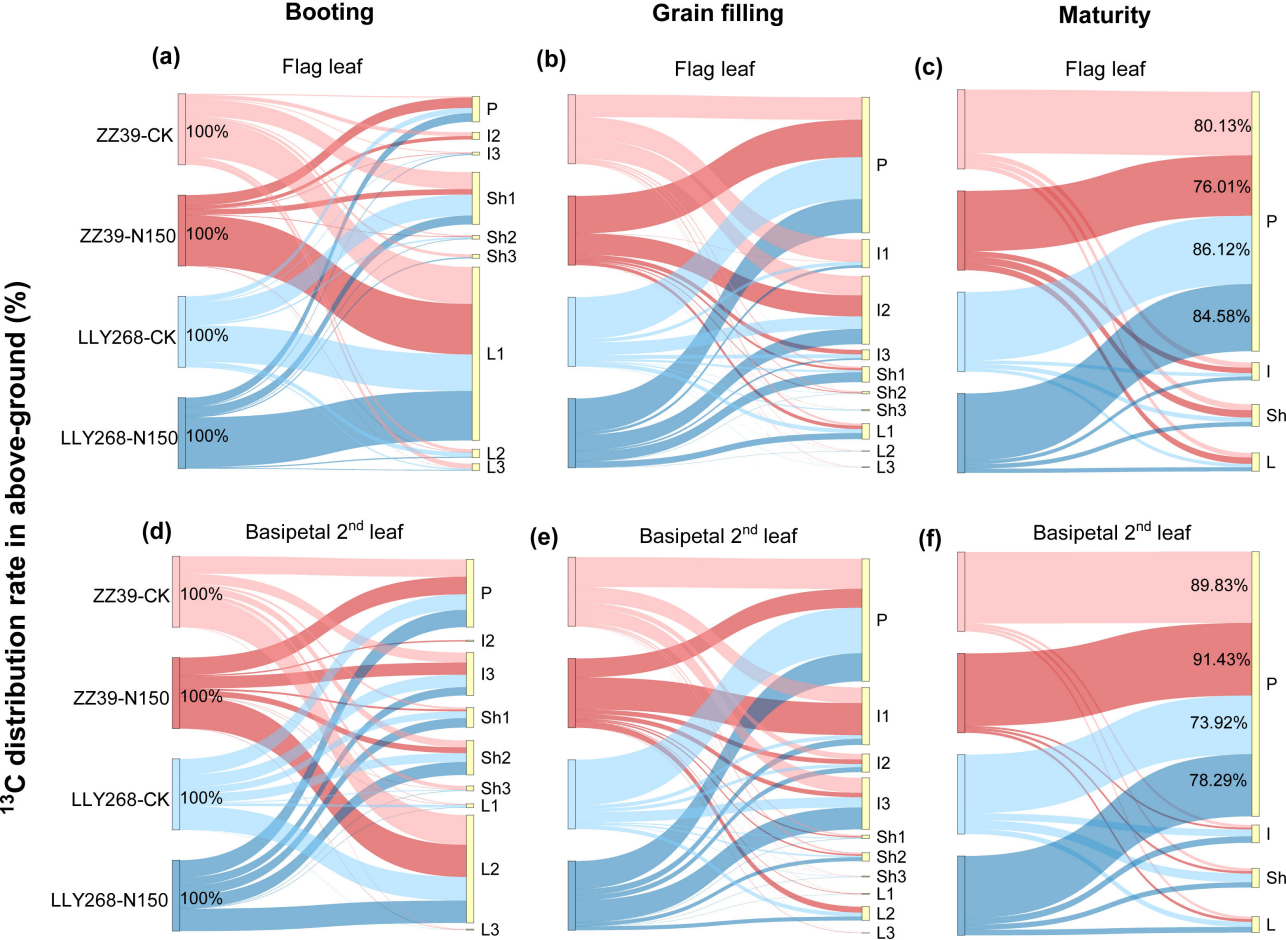
Distribution of photosynthate synthesized by super early-season rice leaves at the booting stage. **(A-C)** Distribution of photosynthates from the booting stage to the maturity stage of the flag leaf, **(D-F)** distribution of photosynthates from the booting stage to the maturity stage of the basipetal 2^nd^ leaf. Note: L1: flag leaf, L2: basipetal 2^nd^ leaf, L3: basipetal 3^rd^ leaf; Sh1: flag sheath, Sh2: basipetal 2^nd^ sheath, Sh3: basipetal 3^rd^ sheath; I1: flag internode, I2: basipetal 2^nd^ internode, I3: basipetal 3^rd^ internode. L: leaf, Sh: sheath, I: internode, P: panicle. The flag internode was not elongated at T1, so no data were available, and the uppermost internode of this stage is I2. Booting stage samples were obtained on June 12^th^ to June 18^th^, the day of the ^13^CO_2_ labeling, grain filling stage samples were obtained on June 27^th^, maturity stage samples were obtained on July 17^th^. 3 replications were measured for each treatment.

During the booting stage, after the assimilation of ^13^CO_2_ and its conversion into photosynthate by leaf photosynthesis, the assimilated carbon were mainly allocated to leaves for leaf growth and maintenance of leaf function (**Figures 9A, D**). Afterwards, photosynthate was distributed from leaves to sheaths and internodes, and finally to panicle and other organs (**Figures 9B, C, E, F**).

During the booting stage, almost 61.1% of the photosynthate in the flag leaves were used directly within the flag leaves under both treatments. The remaining photosynthate was allocated to the panicle through the basipetal first sheath and the basipetal 2^nd^ internode. The rate of photosynthate export from the flag leaves was higher under the CK treatment in comparison to the N150 treatment (**Figure 9A**). On average, 48.2% of the photosynthates from the two cultivars were transported out of the flag leaves under CK treatment, compared to just 29.6% after the N150 treatment at the booting stage (**Figure 9A**). Meanwhile, the photosynthate of basipetal 2^nd^ leaves of both cultivars were distributed in the basipetal 2^nd^ leaves by an average of 38.05% under both treatments (**Figure 9D**). The remaining photosynthate was allocated to the panicle through the basipetal 2^nd^ sheaths and basipetal 3^rd^ internodes. The rate of transfer of photosynthetic products out of the basipetal 2^nd^ leaves was higher under the CK treatment compared to the N150 treatment. In the CK treatment, approximately 48.2% of the photosynthetic products from both cultivars were transported out from the basipetal 2nd leaves. However, under the N150 treatment, only 29.6% of the photosynthetic products were transferred out (**Figure 9D**). During the grain filling stage, around 94.4% of the photosynthetic products from the flag leaves of both cultivars under both treatments were exported from the flag leaves. The majority of these photosynthate were allocated into the basipetal 2nd internode (24.56%), followed by the flag internode (10.2%), and the panicle (48.94%) (**Figure 9B**). In addition, approximately 93.18% of the photosynthate originating from the basipetal 2nd leaves were exported out. The distribution of these products was mostly concentrated in the basipetal 3^rd^ internode (18.6%), basipetal 2nd internode (6.3%), flag internode (20.7%), and panicle (44.2%) (**Figure 9E**). At the maturity stage, almost 81.71% of photosynthate in the flag leaves of both cultivars was transported to the panicle under both conditions. In the case of ZZ39, the average of CK and N150 treatments showed that 78.07% of the photosynthate was transported to the panicle. In contrast, the LLY268 treatments had a higher transfer rate of 85.35% (**Figure 9C**). In addition, about 83.37% of the photosynthate from the basipetal 2nd leaves in two variations were transported to the panicle. In the case of the ZZ39, the transfer rate was 90.63% on average for both CK and N150 treatments. For the LLY268, the transfer rate was 76.11% (**Figure 9F**).

**Figure 10:**
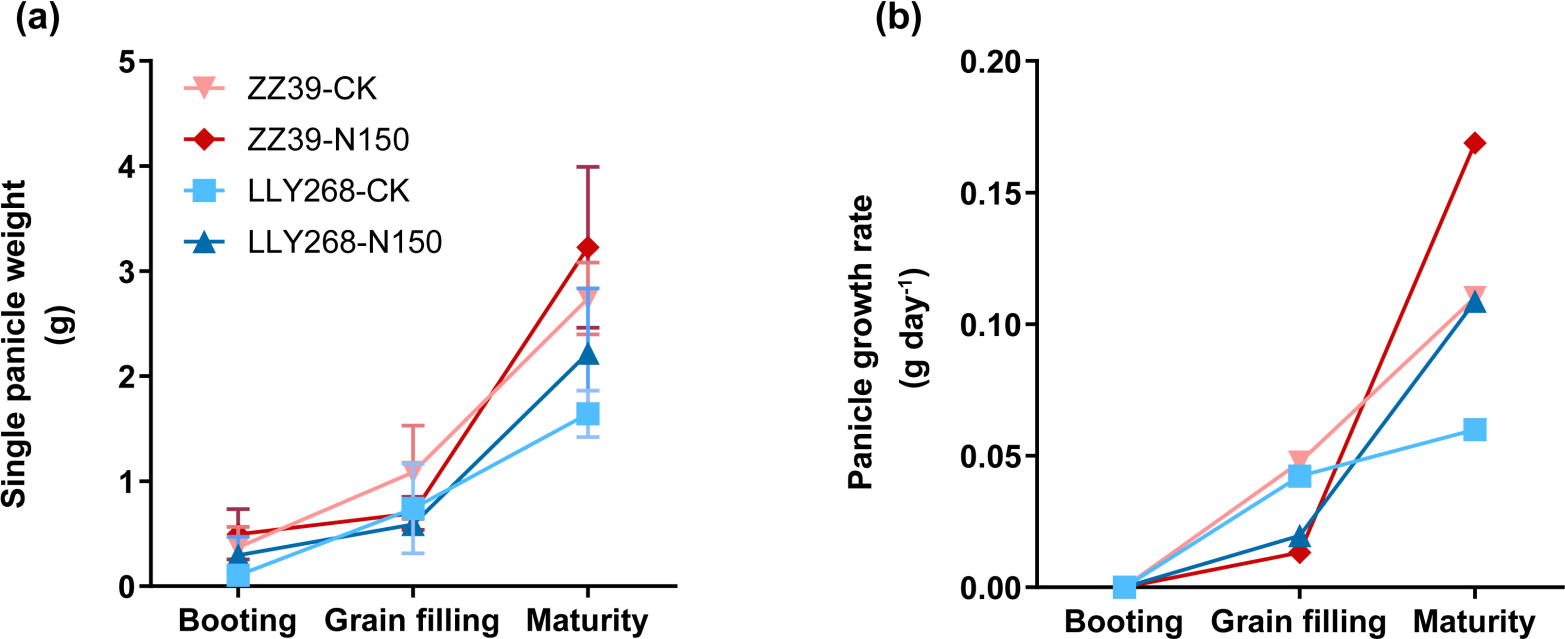
Single panicle growth of super early-season rice. **(A)** dry matter weight of single panicle, **(B)** e growth rate. Booting stage samples were obtained on June 15^th^, grain filling stage samples were obtained on June 27^th^, maturity stage samples were obtained on July 17^th^. Seven replications were measured for each treatment.

We found that the N150 treatment resulted in an increase in the dry matter weight of panicles compared to the control (CK). Specifically, the biomass of single panicles under the N150 treatment increased by 17.88% and 35.37% in the maturity stage of ZZ39 and LLY268, respectively, compared to the control (**Figure 10A**). However, the rate of growth of the panicles of the two cultivars under CK treatment was higher compared to that of N150 from the booting stage to the grain filling stage (**Figure 10B**).

## 4 Discussion

### 4.1 Effects of nitrogen on carbon and nitrogen allocation in a single tiller of an early-season rice

Optimizing N fertilizer application can increase nitrogen use efficiency through enhancing pre-anthesis non-structural carbohydrates accumulation and post-anthesis non-structural carbohydrates translocation (Pan et al., 2016). This non-structural carbohydrates reserved in the stems and sheaths can contribute up to 1/6–1/3 of the grain weight (Okamura et al., 2018) This pattern of increased allocation to the sheath and internode before booting and export of carbon and nitrogen from these tissues after booting were clearly shown in this study (**Figures 6**, **8**). Remarkably, before the booting stage, rice accumulates nitrogen compounds absorbed from the soil in their vegetative organs. However, after the booting stage, nitrogen is mostly allocated to the grain (Ishikawa et al., 2023) with around 80% of nitrogen in the grains is from aging tissues (Ji et al., 2023). Nitrogen application, interestingly, had little effect on the carbon or nitrogen content of different tissues, but it did enhance the nitrogen content of leaves.

In this study, we found that the basipetal 3^rd^ internode had a greater ability for carbon and nitrogen accumulation and transport compared to the upper internodes, consistent with previous findings (Wakabayashi et al., 2022). Nonetheless, the accumulation of carbon and nitrogen in leaves and sheaths differed from that in the internode. The flag leaves and sheath exhibited greater nitrogen buildup than the lower organs (**Figure 6**). However, the carbon accumulation demonstrated that the basipetal 2^nd^ leaves and sheaths displayed enhanced performance (**Figure 8**). These results correspond with the findings of Liang’s study (Liang et al., 2014).

### 4.2 Different patterns of translocation of photosynthate from leaves to other tissues in different rice cultivars

The efficiency of photosynthesis and rice yield are determined by functional leaves, in particular the flag leaves and basipetal 2^nd^ leaves. Previous studies show that significant differences in leaf morphology and photosynthetic properties between the flag leaf and the basipetal 2^nd^ leaf; there is also difference in these properties caused by different leaf positions (Gao et al., 2021; Ding et al., 2022). At the stage of panicle initiation, the leaf area and SPAD of leaves at different leaf positions varied between two cultivars, specifically, the basipetal 2^nd^ leaves had larger values compared to the flag leaves (**Figures 3**, **4**).

**Figure 11:**
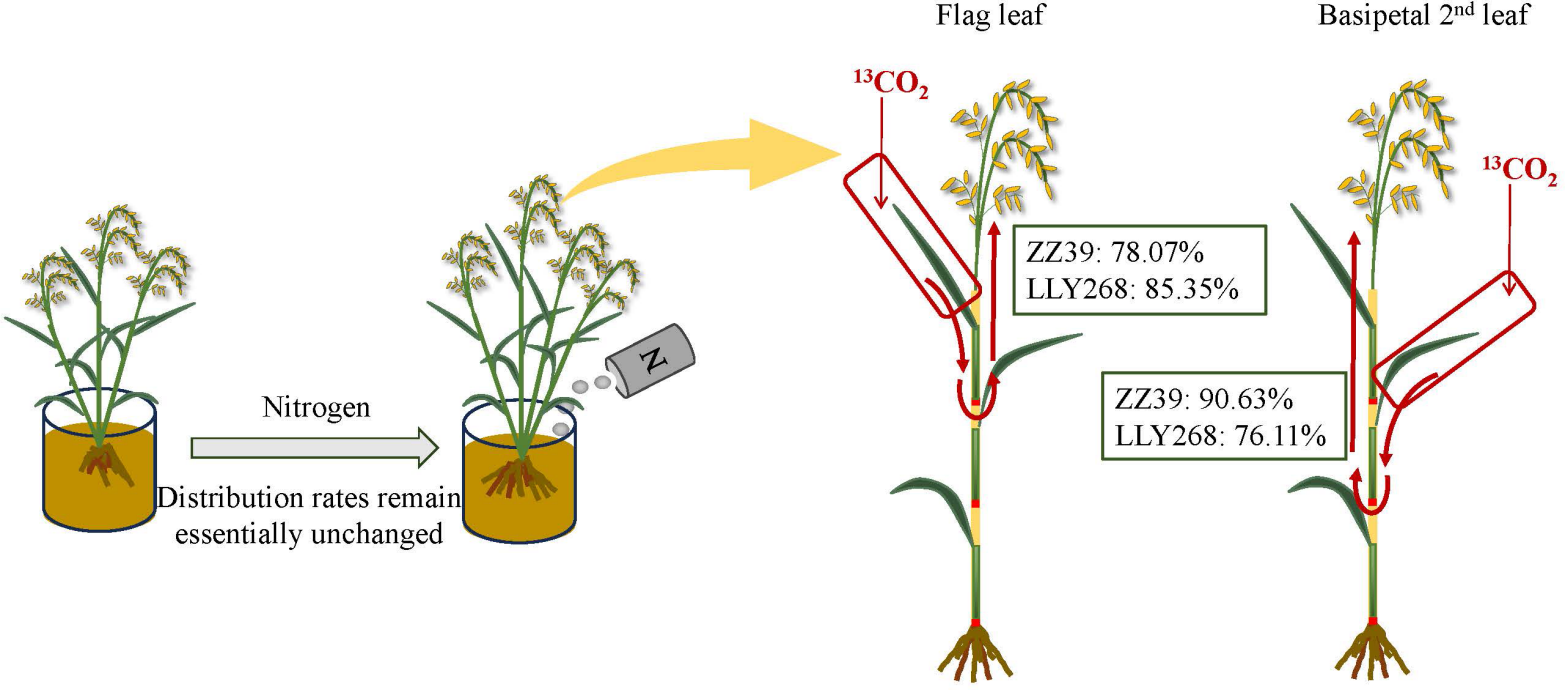
Translocation pathways and mean partitioning rates of leaf photosynthates to the panicle under two nitrogen treatments. The figure shows the distribution and movement of photosynthates from leaves to the panicle, comparing the effects of different nitrogen treatments on partitioning rates.

There are also significant difference in the ability of leaves at different stem positions to gain formation. At the booting stage, after labeling the leaves at different stem positions with ^13^CO_2_, we found a significant proportion (ranging from 73.92% to 91.43%) of the photosynthate from flag leaves and basipetal 2^nd^ leaves of different cultivars were allocated to the panicles. Leaves differentially contribute to grain formation in different cultivars. For example, a greater proportion of photosynthate was allocated to grain at maturity from the basipetal 2^nd^ leaf compared to the flag leaf in ZZ39. In contrast, the difference in this proportion between the flag leaf and basipetal 2^nd^ leaf was relatively minor in LLY268 (**Figures 9**, **11**). During the process of photosynthate transferring, photosynthate from the basipetal 2^nd^ leaf was transferred out of the leaf earlier compare to that of the flag leaf (**Figures 9A, D**), as a result of the difference in the maturity of the flag leaf and the basipetal 2^nd^ leaf.

The observed allocation pattern of photosynthate from this study on early-season rice differs from those on single-season rice (Mohapatra et al., 2004), where the majority of the photosynthate from the upper three leaves during the booting stage were stored in the stems until maturity. This maybe is due to the fact that in single-season rice, which usually has a longer period of growth and a higher biomass, the photosynthate continues to be supplied to the vegetative tissue during the booting period. On the other hand, in early-season rice, which has a shorter growth period and a smaller biomass, the vegetative tissues are mostly developed before the booting stage due to the relatively short growth cycle (Li et al., 2023). As a result, the formed photosynthate can be allocated directly to the panicle, instead of first storing them in the sheath and stem. Interestingly, in low-yielding traditional rice, the flag leave is also primarily responsible for providing carbon assimilates to the grain while in the super high-yielding rice cultivar, e.g. Takanari, the contribution of the basipetal 2^nd^ leaf to grain formation is comparable to that of flag leaves (Mohapatra et al., 2004). These increased number of leaves contributing to grain formation might reflect the enhanced source capacities of these high-yielding cultivars. In the present experiment, the contributions of the flag leaf and the basipetal 2^nd^ leaf to the grain of both cultivars were similar, which is similar to the pattern of photosynthate allocation in high-yielding rice. There were deficiencies in this experiment in that the ^13^CO_2_ labeling experiment was conducted for only one year, and because of the difficulty of labeling in the field, a field trial was not conducted. However, field experiments were carried out previously (Wang et al., 2021), and the data on yield composition and other data from this pot experiment are reliable.

### 4.3 Flexibility in the responses of allocation patterns to nitrogen treatments in rice cultivars with different yield components

After nitrogen application, the yield of ZZ39 increased by 90.42% and the yield of LLY268 increased by 72.61% (**Table 2**). Our finding is consistent with the earlier finding that the response to nitrogen application is more pronounced in cultivars with larger panicles (Liao et al., 2023). Remarkably, though nitrogen application increased rice yield dramatically in both cultivars; however, the increases in yields were achieved through complexly different mechanisms. Specifically, following the application of nitrogen fertilizer, ZZ39 showed increased number of effective panicles, whereas LLY268 showed increased number of grains per panicle (**Table 2**). These responses reflect, in some sense, an optimal response of rice to nitrogen application. Prior field experiments have revealed variations in the yield components’ response to nitrogen between the two cultivars. Notably, at a nitrogen application of 150 kg·hm^-2^, the yield increases for both cultivars were similar, averaging 27.51%. However, the increase in the number of effective panicles was greater for ZZ39 compared to LLY268 regarding yield components (Wang et al., 2021). These differential responses in yield components in these two rice cultivars are underlined by their different patterns of carbon and nitrogen under nitrogen treatment (**Figures 6**, **8**, **9**). Such near-optimal responses in these allocation patterns might reflect the artificial selection by breeders for cultivars which can adjust their growth and development patterns to gain superior yield in a relatively short growth cycle. For instance, LLY268 exhibits a greater number of effective panicles, necessitating increased transport of photosynthates from individual stems to other tillers. This is evidenced by the observation that photosynthates from the basipetal 2^nd^ leaf were more frequently transported to the lower internode compared to ZZ39 during the filling stage (**Figure 9E**). Concurrently, the C content indicated that the lower internode of LLY268 had a more pronounced rise during the filling stage (**Figure 8C**).Therefore, early-season rice might be used as a model to study how plants gain flexible and tailored responses to nitrogen treatment to gain higher yields.

Besides these tailored and near-optimal responses of photosynthate allocation under nitrogen treatment, we also found that the speed of photosynthate translocation was slower (**Figures 8**, **9**), and the panicle growth rates were slower at the beginning of grain filling as well (**Figure 10**) under nitrogen treatment. This finding is consistent with earlier findings that α-amylase and β-amylase were more efficient in the rice stalk under low nitrogen conditions (Li et al., 2018a, 2022), the higher rate of NSC translocation in sheaths under low nitrogen (Zakari et al., 2020) and constant ^13^C distribution ratio in rice under nitrogen application (Xiao et al., 2019).

## 5 In conclusion

This study examined the alterations in the rate of translocation of photosynthate into different organs including sheath, internode, and grains during the maturity stage in two early-season rice lines under normal and increased nitrogen application. We found that in the early-season rice, the photosynthate of the flag leaf and also the basipetal 2^nd^ leaf are mostly allocated directly to support grain formation, instead of storing them in the stem and sheath. In addition, in cultivars with different panicle size or the number of effective panicles, nitrogen application showed different impacts on the panicle size and effective panicle number. Such differential impacts on the yield components are also reflected in the changes in the allocation patterns of carbon and nitrogen under nitrogen application. Such near-optimal responses of early-season rice to nitrogen fertilizer application might be a result of long-term artificial selection, where breeders unintentionally selected those lines that can optimally adjust their growth and development to different environments, in particular, soil nitrogen status, to gain optimal yield. Therefore, early-season rice cultivars might be used as model plants to study the flexibility of plant response strategies to cope with external environment changes.

## Data Availability

The original contributions presented in the study are included in the article/supplementary material. Further inquiries can be directed to the corresponding authors.
